# Metastatic Thyroid Cancer Presenting as Renal Cortical Mass

**DOI:** 10.1155/2020/9816479

**Published:** 2020-01-06

**Authors:** Osamah Hasan, Matthew Houlihan, Tobias S. Kohler, Courtney M. P. Hollowell

**Affiliations:** ^1^Midwestern University Chicago College of Osteopathic Medicine, Downers Grove, IL, USA; ^2^Department of Urology, Mayo Clinic, Rochester, MN, USA; ^3^Division of Urology, Cook County Health and Hospitals System, Chicago, IL, USA

## Abstract

The authors present a rare case of primary diagnosis of metastatic, differentiated thyroid cancer presenting as a solitary, large renal mass. Renal cortical masses which represent metastatic primary malignancies are often small, multifocal, and in the setting of active malignancy. Surgical excision of this patient's renal mass demonstrated the unexpected diagnosis and subsequent endocrine surgical intervention.

## 1. Introduction

The presentation, diagnosis, and treatment of renal cortical masses, has evolved substantially over the last forty years. Historically, the standard of care has been surgical excision via partial or radical nephrectomy. Traditional therapy has evolved in the modern era due in large part to increasing incidence and earlier diagnosis of renal masses, which is due in large part to increased utilization of cross-sectional imaging in health care [1]. Management options of cT1 renal masses may include active surveillance, thermal ablation, or extirpation via partial or radical nephrectomy depending on the size and location of the tumor, as well as patient-specific factors [2]. We report a unique urologic case with a primary diagnosis of differentiated thyroid cancer after undergoing nephrectomy for a cT1bN0M0 renal mass.

## 2. Case Report

### 2.1. Patient Presentation

The patient was a 64-year-old female with a past medical history of hypertension, diabetes mellitus type 2, and hyperlipidemia referred to urology clinic for evaluation of a 5 cm left-sided renal mass that was incidentally discovered during evaluation for diarrhea and flank pain. She denied any history of gross hematuria or other urinary symptoms. She had no significant past medical history. The patient had never smoked and denied a family history of renal malignancy. On physical examination, she was found to be in no acute distress, and appeared generally well (ECOG performance status 0). There were no carotid bruits or neck masses palpated. Her chest was clear to auscultation bilaterally and her abdomen was soft, nontender, and nondistended without palpable abdominal masses or costo-vertebral angle tenderness. Laboratory evaluation including comprehensive metabolic panel, complete blood count, and urinalysis were within normal limits.

### 2.2. Imaging Studies

**Figure 1:**
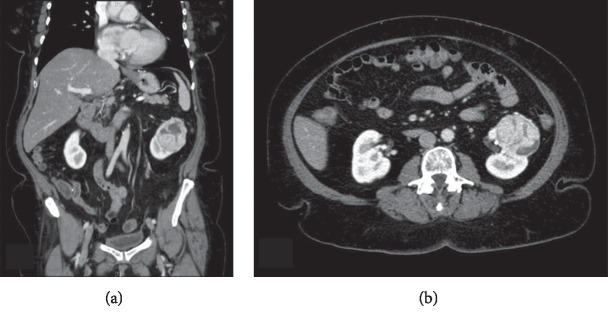
CT A&P demonstrating a 4.9 cm × 5.0 cm × 5.8 cm renal mass. Coronal image (a), axial (b).

**Figure 2:**
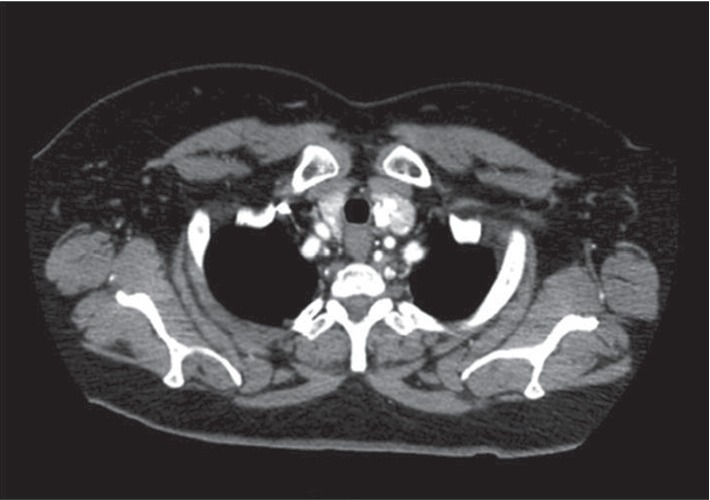
CT chest w/contrast demonstrating multinodular goiter.

Computed tomography of the abdomen and pelvis with contrast demonstrated a 4.9 cm by 5.0 cm by 5.8 cm endophytic interpolar left renal mass (Figures 1(a) and 1(b)). Chest CT with contrast demonstrated a 12 mm right lower lobe pulmonary nodule, which was noted to have been stable on prior imaging up to eleven years previously and was interpreted as a granuloma. Finally, she was found to have a slightly enlarged thyroid gland with several small calcified nodules (Figure 2).

### 2.3. Surgical Management

The patient was counseled regarding options for management and underwent hand-assisted laparoscopic radical nephrectomy. The surgical procedure was uncomplicated with an estimated blood loss of 25 cc. She tolerated surgery well, and, following an uncomplicated recovery, was discharged from the hospital on postoperative day three.

### 2.4. Patient Outcome

**Figure 3:**
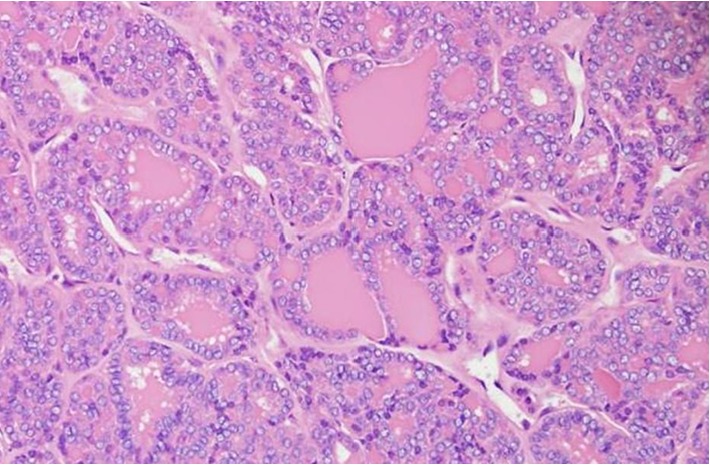
Photomicrograph showing follicular variant of papillary thyroid carcinoma (H&E stain, 100x).

Histopathology revealed a 6.5 cm mass consistent with metastatic follicular variant of papillary thyroid cancer (Figure 3). The diagnosis was confirmed with immunohistochemistry as the tumor cells were CK7 (+), TTF1 (+), and Thyroglobulin (+) but CK20 (−) and WT1 (−). The soft tissue and vascular surgical margins were negative for tumor. Given the results of the pathologic analysis and evidence of several calcified nodules within the enlarged thyroid, she was referred to endocrine surgery for further evaluation. On thyroid ultrasound, a left-sided thyroid nodule was appreciated. Her endocrine evaluation included thyroid function panel, which demonstrated an elevated thyroid-stimulating hormone (TSH) of 7.57 uIU/mL and normal T4 (0.81 ng/dL). She underwent fine-needle-aspiration of the thyroid, which showed atypia. Subsequent nuclear medicine whole body thyroid scan demonstrated no additional sites of I-131 avidity. She underwent a total thyroidectomy and radioactive iodine 3 months after. Pathology was consistent with a follicular variant of papillary thyroid carcinoma.

## 3. Discussion

The differential diagnosis for pathologic etiology of cT1bN0M0 renal masses is broad. Renal cortical masses may be either benign or malignant. Malignant renal cortical masses include renal cell carcinoma, metastases from other malignancies and renal medullary carcinoma [3]. Renal cell carcinoma is the most common malignant cause of renal cortical masses and can be divided into histologic subtypes including clear cell, multilocular cystic clear cell, chromophobe, papillary, collecting duct, unclassified, postneuroblastoma, and mucinous tubular and spindle cell carcinoma [4]. Clear cell renal cell carcinoma is the most common histologic subtype representing roughly 75% of malignant renal masses with papillary renal cell carcinoma representing 10–15% [4]. Patients with clear cell typically have a worse prognosis compared with other histologic subtypes [5].

Differentiated thyroid cancer consists of both papillary and follicular carcinomas. Differentiated thyroid cancer typically remains localized to the thyroid and presents with a thyroid mass. Incidence of distant metastatic disease has been studied to be less than 4% [6]. The most common sites of metastatic thyroid cancer are the cervical lymph nodes, lungs, bones, and brain [7]. It may also present with regional lymph node metastasis [8]. There are rare case reports describing metastatic thyroid cancer involving the genitourinary organs which include adrenal glands, kidneys, and penile and scrotal skin [9, 10]. Thyroid cancer is an indolent diagnosis, with overall survival greater than 85% at 20 years when localized to the thyroid gland [11]. The prognosis of well-differentiated thyroid cancer is significantly worsened by distant metastatic disease, with a 47-fold increase in risk of death [11]. Upon review of literature, 25 cases have been described involving metastatic differentiated thyroid cancer to the kidney [12]. Most cases described are in females over the age of 45 years [12]. However, diagnosis of metastatic differentiated thyroid cancer presenting as a primary renal mass has only been described in only two prior cases, per the authors' review [13, 14]. Both patients had no other evidence of metastatic disease and as such underwent uncomplicated nephrectomies with subsequent diagnosis of primary differentiated thyroid carcinoma [13, 14]. Both patients were treated post-nephrectomy with total thyroidectomies, with one patient receiving post-thyroidectomy radioactive iodine. Follow-up data was available for one patient demonstrating freedom from local or distant metastases three years following radical nephrectomy [13]. Nonrenal cell carcinoma cortical metastases from other primary tumors are typically multifocal and bilateral, discovered in the setting of other metastases, and present as small, endophytic masses [15]. Our case discussed represented a solitary lesion, 5.8 cm in greatest dimension, with primarily exophytic components without evidence of other sites of metastatic disease.

## 4. Conclusion

In the setting of an enlarged thyroid gland with calcified nodules during staging imaging, metastatic thyroid cancer should be included in the differential diagnosis of a localized renal mass. In such cases, baseline laboratory evaluation may include thyroid function test and preoperative consultation with endocrine surgery should be considered. Surgical excision of pT1bN0M0 renal mass via radical or partial nephrectomy in an otherwise healthy female remains the standard of care. A multidisciplinary approach to postoperative follow-up and care is essential in the setting of rare and unusual cases such as that presented here.
